# Putative genes in alkaloid biosynthesis identified in *Dendrobium officinale* by correlating the contents of major bioactive metabolites with genes expression between Protocorm-like bodies and leaves

**DOI:** 10.1186/s12864-021-07887-6

**Published:** 2021-07-29

**Authors:** Zhaojian Wang, Weimin Jiang, Yingying Liu, Xiaoxi Meng, Xinglong Su, Mengyang Cao, Liping Wu, Nianjun Yu, Shihai Xing, Daiyin Peng

**Affiliations:** 1 College of Pharmacy, Anhui University of Chinese Medicine, Hefei, 230012 China; 2 Institute of Traditional Chinese Medicine Resources Protection and Development, Anhui Academy of Chinese Medicine, Hefei, 230012 China; 3 Hunan Key Laboratory for Conservation and Utilization of Biological Resources in the Nanyue Mountainous Region, Hengyang Normal University, Hengyang, 421008 China; 4 College of Humanities and International Education Exchange, Anhui University of Chinese Medicine, Hefei, 230012 China; 5 Department of Horticultural Science, University of Minnesota, Minneapolis, MN 55108 USA; 6 Anhui Province Key Laboratory of Research & Development of Chinese Medicine, Hefei, 230012 China; 7 Synergetic Innovation Center of Anhui Authentic Chinese Medicine Quality Improvement, Hefei, 230038 China

**Keywords:** *Dendrobium officinale*, Protocorm-like body, Polysaccharide, Alkaloid, Flavonoid

## Abstract

**Background:**

*Dendrobium officinale*, an endangered Chinese herb, possesses extensive therapeutic effects and contains bioactive ingredients such as major polysaccharides, alkaloids, and minimal flavonoids. We first obtained the protocorm-like bodies (PLBs) of this plant through tissue culture in order to determine the distribution of the main secondary metabolites in each organelle and the PLBs. We then analyzed the correlation between gene expression level from comparative transcriptome sequencing and metabolite content in different organs to identify putative genes encoding enzymes involved in the biosynthesis of polysaccharides, alkaloids, and flavonoids.

**Results:**

We used seeds as explants for protocorm induction and PLB propagation of *D. officinale*. The optimal medium formula for PLB propagation was 1/2 MS + α-NAA 0.5 mg·L^− 1^ + 6-BA 1.0 mg·L^− 1^ + 2, 4-D 1.5–2.0 mg·L^− 1^ + potato juice 100 g·L^− 1^. Stems, PLBs and leaves of *D. officinale* had the highest content of polysaccharides, alkaloids and flavonoids, respectively. Naringenin was only produced in stem; however, PLBs with high alkaloid content can replace other organs producing alkaloids. The hot water extraction method outperformed the ultrasound-assisted extraction method for extracting polysaccharides from *D. officinale.* A comparative transcriptome analysis of PLBs and leaves of *D. officinale* revealed differential expression of genes encoding enzymes involved in polysaccharide, alkaloid and flavonoid biosynthetic pathways. Putative genes encoding enzymes involved in these biosynthetic pathways were identified. Notably, we identified genes encoding the alkaloid biosynthesis enzymes strictosidine β-D-Glucosidase, geissoschizine synthase and vinorine synthase in *D. officinale*.

**Conclusions:**

The identification of candidate genes encoding enzymes involved in metabolite biosynthesis will help to explore and protect this endangered species and facilitate further analysis of the molecular mechanism of secondary metabolite biosynthesis in *D. officinale*.

**Supplementary Information:**

The online version contains supplementary material available at 10.1186/s12864-021-07887-6.

## Background

*Dendrobium officinale* Kimura et Migo, a precious, perennial, epiphytic herb in China and other Asian countries [1], is endangered owing to overexploitation and habitat destruction because of its high medicinal value. The stem of *D. officinale* has been used as a Traditional Chinese Medicine (TCM) since the Tang dynasty about 1300 years ago, its effects are tonifying the stomach, nourishing body fluids, clearing heat, nourishing “yin” and improving immunological function [2]. Furthermore, *D. officinale* plays an important role in treating atrophic gastritis, diabetes, cancer, cardiovascular disease and cataract disease, and in delaying aging [3]. Its bioactive components include major *D. officinale* polysaccharides (DOPs) [4] and alkaloids [5], and minimal flavonoids [6]. DOPs cure hypoglycemia [7], and have antioxidation [8], immunomodulation [9] and anti-tumor effects. Alkaloids of *D. officinale* show antioxidant, anticancer, and neuroprotective activities [10], while flavonoids of *D. officinale* possess good anti-cytotoxicity and antioxidant functions [11].

Polysaccharides are the major medicinal components of *D. officinale* [12], and their biosynthesis includes three main stages in medicinal plants [13, 14]. Firstly, sucrose produced by photosynthesis is converts into two types of monosaccharides—glucose 1-phosphate (Glc-1P) and fructose-6-phosphate (Fru-6P)—via a series of enzymatic reactions. Secondly, these two monosaccharides are extended into various nucleotide-diphospho-sugars (NDP-sugars, with NDP including UDP and GDP) by enzymatic reactions. Finally, all of the NDP-sugars are transformed into macromolecular polysaccharides through successive catalysis by glycosyltransferases (GTs) [15]. The biosynthetic pathway of DOPs was predicted using the KEGG database as shown in Additional file 1 [16, 17]. NDP-sugars can be turned into many types of DOP in the *D. officinale* Golgi complex, catalyzed by different kinds of glycosyltransferases such as glucosyltransferases, fucosyltransferases, mannosytransferases and xylosyltransferases [12, 18]. Water-soluble polysaccharides, mostly mannose and to a lesser extent glucose, are the dominant DOPs in *D. officinale* stems [19]. Mannan polysaccharide is catalyzed by mannan synthases, which are encoded by members of the *Cellulose synthase-like A* (*CslA*) family [20, 21] (Additional file 1).

Alkaloids are another major medicinal component of *D. officinale*, most of which are terpenoid indole alkaloids (TIAs) [22]. Many types of TIAs are formed from a strictosidine backbone precursor [23], representing a conserved upstream biosynthetic pathway for TIAs in plants producing various specific downstream alkaloids [24]. Moreover, there might be a series of P450 monooxygenases and aminotransferases following strictosidine in the downstream biosynthetic pathway of TIAs [25]. Cytochrome P450s are involved in oxidation and hydroxylation reactions, while aminotransferases transfer amino acids to form alkaloids, which are nitrogenous amino acid derivatives [26]. A *polyneuridine-aldehyde esterase* (*PNAE*) gene participates in 16-epivellosimine biosynthesis in *D. officinale* [12]. Therefore, based on the KEGG database and chemical structure of the compounds, we proposed a putative alkaloid biosynthetic pathway of *D. officinale* (Additional file 2) [18, 22, 27]. In this downstream alkaloid biosynthetic pathway, strictosidine is catalyzed to strictosidine aglycone by β-D-glucosidase (SG). The conversion of strictosidine aglycone to 4,21-dehydro-geissoschizine is catalyzed by successive unknown enzymes, and 4,21-dehydro-geissoschizine is reciprocally catalyzed to geissoschizine by another unknown enzyme. We predict that vinorine might be one of the alkaloids in *D. officinale*, biosynthesized from 16-epivellosimine via the catalysis of vinorine synthase (VS).

Flavonoids all originated from two intermediates (L-tyrosine and L-phenylalanine) produced by the shikimate pathway in plants, which generates p-coumarinyl-CoA via enzyme catalysis of p-cinnamic acid and cinnamoyl-CoA, respectively [28]. Different types of flavonoids, such as flavone, flavanone, flavonol and anthocyanin, are then produced by various enzymatic reactions from p-coumaryl-CoA [29]. Among these flavonoids, naringenin is important and has many clear effects on the human body, such as inhibiting the inflammatory response of many cell types [30] and having hypotensive [31], anti-fibrotic and anti-cancer as well as hepatoprotective effects [32]. Chalcone isomerase (CHI) catalyzes naringin chalcone to naringin, which is condensed from three malonyl-CoAs and one p-coumaryl-CoA via chalcone synthase (CHS) [33]. The putative biosynthesis pathway of flavonoids in *D. officinale* is shown in Additional file 3 [34].

Wild *D. officinale* is endangered and on the IUCN Red List of Threatened Species [34]. Therefore, it is imperative to conserve wild resources of *D. officinale* and seek alternative ways to propagate it. Tissue culture technology may be a suitable method for *D. officinale* breeding via the induction of protocorms from embryogenic tissues. Protocorm-like bodies (PLBs) from non-embryogenic tissues of *D. officinale* have been induced [35]; however, the content of the main bioactive compounds in *D. officinale* PLBs varies with medium formulae and the elicitors used as additives [36].

This study aimed to establish a system for protocorm and PLB induction and proliferation for *D. officinale*. We compared the contents of main metabolites such as polysaccharides, alkaloids, flavonoids and naringenin (one of the special flavonoids) in PLBs and organs from the original plant. There were significant differences in contents of these metabolites between PLBs and plant organs, suggesting that PLBs and leaves were good for analyzing the expression levels of candidate enzyme-encoding genes involved in the specific biosynthetic pathways of these metabolites in *D. officinale*. Based on the differential expression of genes (DEGs) from transcriptome sequencing, we identified or verified putative genes encoding enzymes involved in biosynthetic pathways of the main metabolites. This study will help to conserve *D. officinale* resources and more deeply understand and study the biosynthesis and regulation of its main metabolites.

## Results

### Protocorm and PLB induction of *D. officinale*

We screened the optimal explants for PLB induction and the optimum formula for PLB propagation in order to construct a comprehensive system for PLB proliferation and protocorm/PLB induction for *D. officinale*. Induction rate and the growth status were determined among the five explants: capsules approaching maturity, leaves, stem tips, stem fragments and stems with nodes. Seeds was the most suitable material for protocorm of PLB induction, and capsules approaching maturity were collected and punctured to release seeds under sterile condition because of the tiny seeds (Table 1).

**Table 1:** Effects of different explants on protocorms and PLBs induction

Explants	Seeds	Leaves	Stem fragments	Stem tips	Stem with nodes
Induction rate (%)	100 ± 0.0	43 ± 0.9	25 ± 1.8	52 ± 1.6	31 ± 1.9
Growth status	Well	Common	Not good	Common	Common

**Fig. 1:**
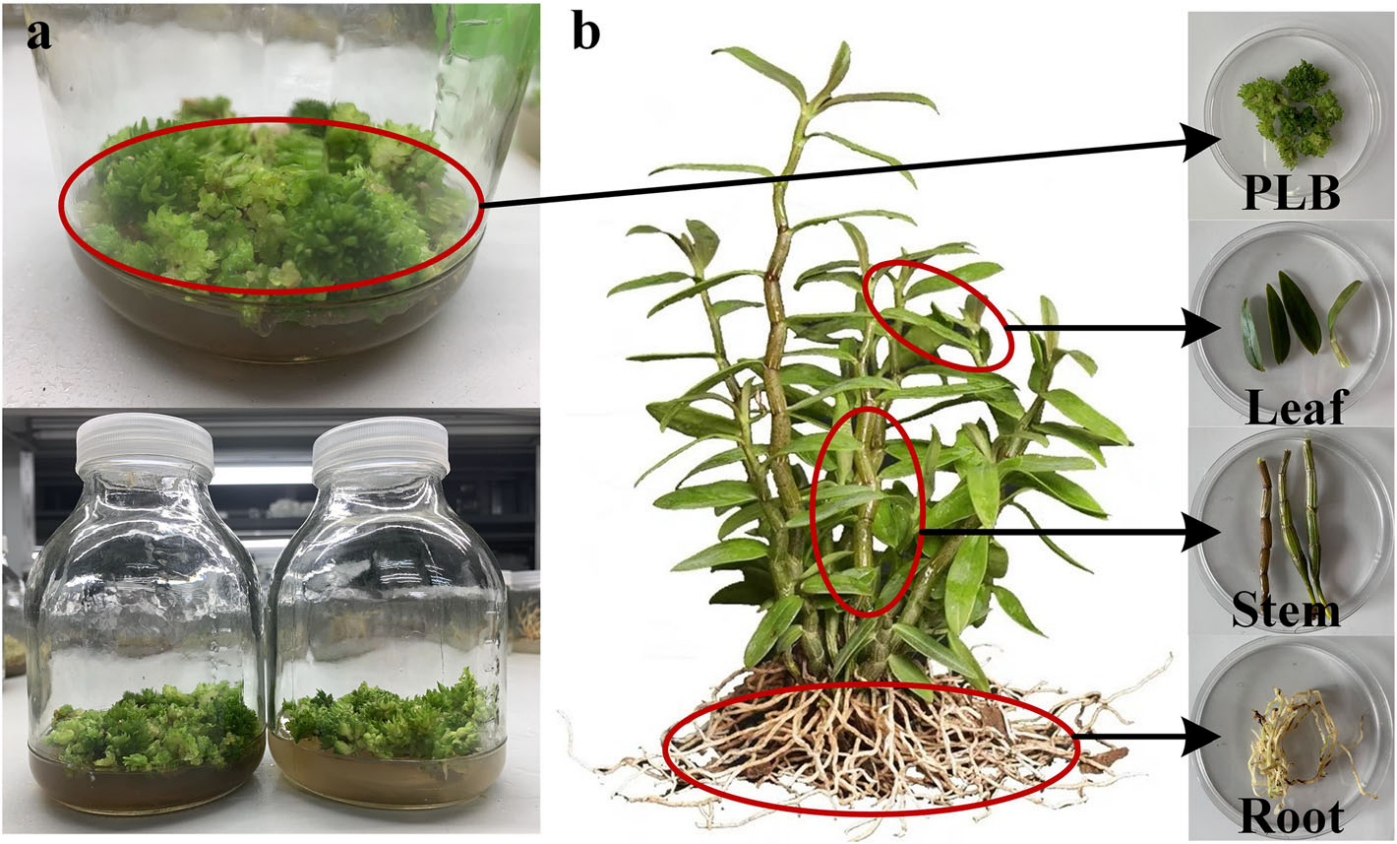
Determination of polysaccharide, alkaloid and flavonoid contents in three different organs of *D. officinale* and protocorms. **a** PLBs of *D. officinale* obtained by tissue culture. **b** Samples from different parts of *D. officinale*

An L_16_(4^5^) orthogonal experiment was designed for screening the best protocol for PLB propagation of *D. officinale*. The best formula was A3B3C2D1(2)E2, with factor C (6-BA [6-benzylaminopurine] content) being the most important factor because of its high R value. A verification experiment with formula A3B3C2D2E2 was conducted with 40 replicates, and the propagation coefficient was up to 4.37 (Table 2, Fig. 1 a). The optimal formula for PLB propagation of *D. officinale* was 1/2 MS + α-NAA (α-naphthylacetic acid) 0.5 mg·L^− 1^ + 6-BA 1.0 mg·L^− 1^ + 2, 4-D (2,4-dichlorophenoxyacetic acid) 1.5–2.0 mg·L^− 1^ + potato juice 100 g·L^− 1^.

**Table 2:** The effects of different factors on PLBs proliferation

Levels		Factors				Propagation coefficient
	Media (A)	α-NAA (B) mg·L^−1^	6-BA (C) mg·L^− 1^	2,4-D (D) mg·L^− 1^	Potato juice (E) g·L^− 1^	
1	1/4MS	0.1	0.5	1.5	50.0	2.14
2	1/4MS	0.2	1.0	2.0	100.0	3.98
3	1/4MS	0.5	1.5	2.5	150.0	3.23
4	1/4MS	1.0	2.0	3.0	200.0	1.98
5	1/2MS	0.1	1.0	2.5	200.0	4.54
6	1/2MS	0.2	0.5	3.0	150.0	1.83
7	1/2MS	0.5	2.0	1.5	100.0	4.57
8	1/2MS	1.0	1.5	2.0	50.0	3.11
9	MS	0.1	1.5	3.0	100.0	3.42
10	MS	0.2	2.0	2.5	50.0	2.75
11	MS	0.5	0.5	2.0	200.0	4.63
12	MS	1.0	1.0	1.5	150.0	5.02
13	2MS	0.1	2.0	2.0	150.0	2.65
14	2MS	0.2	1.5	1.5	200.0	1.93
15	2MS	0.5	1.0	3.0	50.0	4.87
16	2MS	1.0	0.5	2.5	100.0	2.53
K1	2.83	3.19	2.78	3.60	3.22	
K2	3.51	2.62	4.60	3.59	3.62	
K3	3.96	4.32	2.92	3.26	3.18	
K4	3.00	3.16	2.99	3.02	3.27	
R	1.13	1.16	1.82	0.52	0.44

### Total content of polysaccharides, alkaloids and flavonoids in PLBs and organs of *D. officinale*

**Fig. 2:**
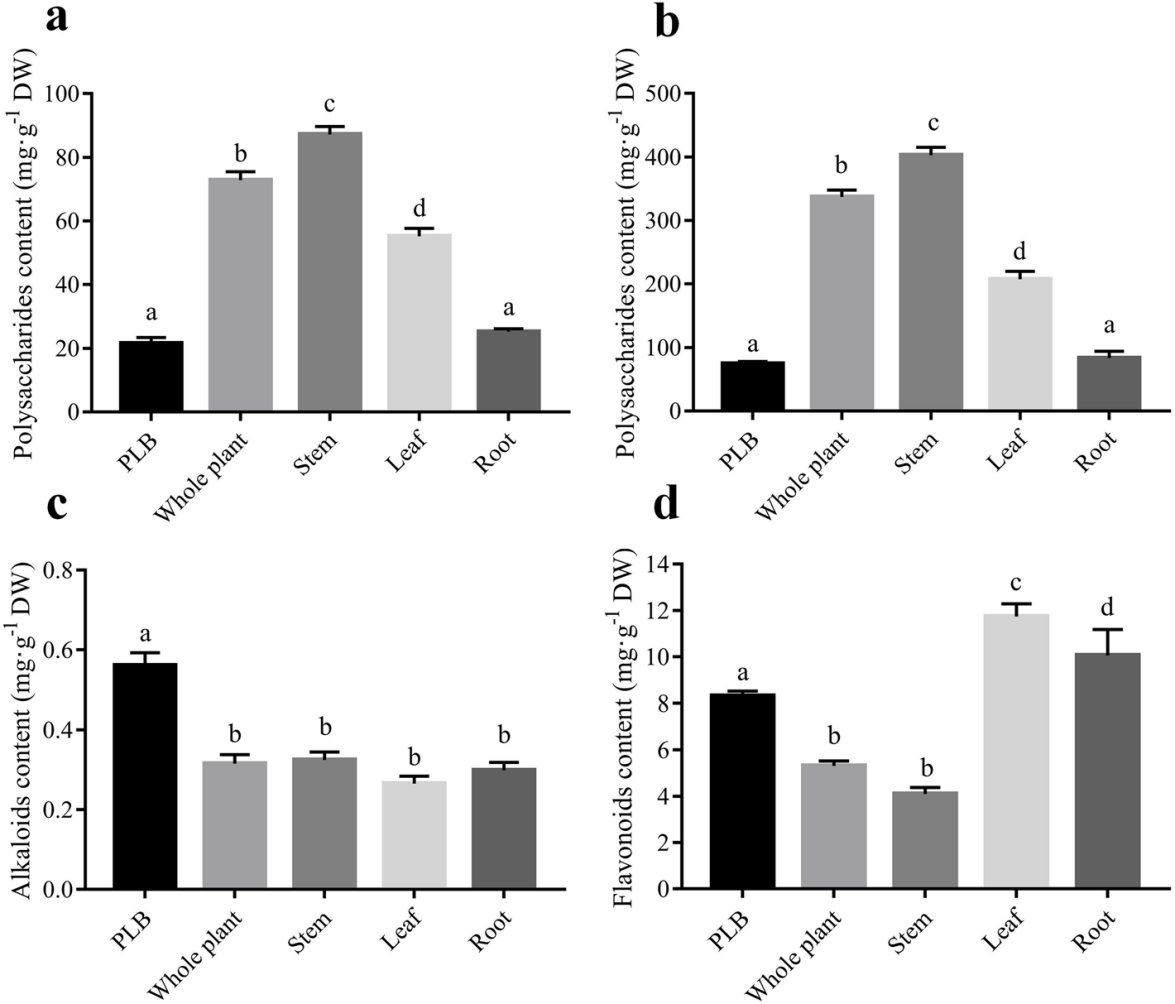
Determination of the content of polysaccharides, alkaloids and flavonoids in PLBs and different organs of *D. officinale*. **a** Contents of total DOPs in PLBs and different organs using the hot water extraction (HWE) method. **b** Contents of total DOPs in PLBs and different organs using the ultrasound-assisted extraction (UAE) method. **c** Contents of total alkaloids in PLBs and different organs. **d** Contents of total flavonoids in PLBs and different organs. DW, dried weight of samples. Different letters associated with bars within the same histogram represent significant differences at *p* ≤ 0.05 or 0.01

We determined contents of polysaccharides, flavonoids and alkaloids in various organs of *D. officinale*, including the whole plant, root, stem, leaf and PLB (Fig. 1 b). Significant differences in contents of these three components among samples were detected by variance analysis and multiple comparisons (Fig. 2).

#### Differential contents of Total polysaccharides (DOPs)

The regression equations for the D-glucose standard curves obtained using ultrasound-assisted extraction (UAE) and hot water extraction (HWE) methods of polysaccharide extraction were Y = 0.0491X -0.0035 (R^2^ = 0.9997) and Y = 0.0684X - 0.0323 (R^2^ = 0.9998) at 488 nm, respectively (Additional file 4). Contents of polysaccharides in PLBs and organs measured using two different extraction methods showed the same content distribution, but the extraction rate using the HWE method was higher than that using the UAE method (Fig. 2 a and b). We therefore concluded that the HWE method was better for DOP extraction.

Distributions of DOPs in plant organs were as follows: stem > whole plant > leaf > root. The stem of *D. officinale* had the highest content of polysaccharides among all organs at 402.93 mg·g^− 1^ DW (Dried weight). While, PLBs had a lower content of polysaccharides than plant organs with average amount of 75.30 mg·g^− 1^ DW (Fig. 2).

#### Differential contents of Total alkaloids

The equation for the standard curve using dendrobine as reference was Y = 0.1154X-0.0048 (R^2^ = 0.9990), with absorbance detected at 620 nm (Additional file 4). We observed no significant differences in total alkaloid contents among different parts of *D. officinale* by multiple comparisons [all around 0.3 mg·g^− 1^ DW]. Meanwhile, the total alkaloids contents in PLBs ranged up to 0.56 mg·g^− 1^ DW, which was almost twice as high as that of *D. officinale* plant organs (Fig. 2 c). PLBs can therefore be used as alternative materials for dendrobine extraction.

#### Differential contents of Total flavonoids and Naringenin

The regression equation for the standard curve for total flavonoids measured using rutin as the reference was Y = 0.0114X-0.0009, with R^2^ = 0.9992 and absorbance is at 510 nm. *D. officinale* stems had the lowest content of total flavonoid, with only 4.07 mg·g^− 1^ DW, which was consistent with most findings reported previously [34]. Leaf had the highest content of total flavonoids at 11.74 mg·g^− 1^ DW followed by root (Additional file 4). The total flavonoids content in PLBs was higher than that in the whole plant and stem, but lower than that in the leaf and root (Fig. 2 d). Considering the low biomass of leaf and root in *D. officinale*, PLBs would be the most suitable materials for extracting total flavonoids of *D. officinale*.

**Fig. 3:**
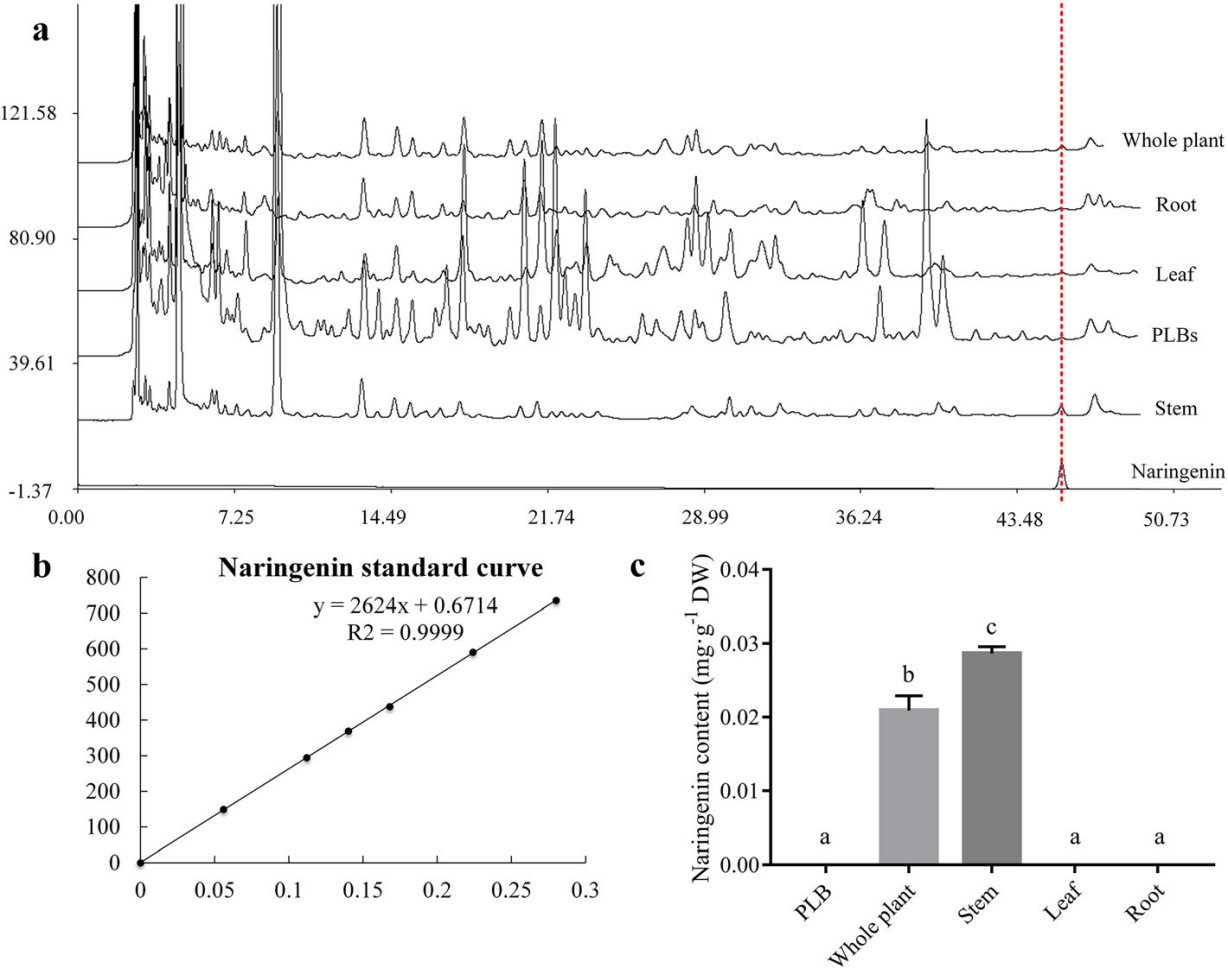
Determination of the naringenin. Content in *D. officinale*. **a** HPLC-UV chromatogram of *D. officinale* samples and naringenin standard. **b** Standard curve of naringenin; X-axis represents naringenin content, and Y-axis represents peak area. **c** Content of naringenin in PLBs and different organs of *D. officinale*. DW, dried weight of the samples. Different letters associated with bars within the same histogram represent significant differences at p ≤ 0.05 or 0.01

The content of naringenin, an important flavonoid was determined by HPLC-UV, and the chromatogram showed a retention time (RT) was at 44.8 min (Fig. 3 a). The regression equation of the standard curve for naringenin was Y = 2624X + 0.6714, R^2^ = 0.9999 (Fig. 3 b). Naringenin could only be quantified in the stems of *D. officinale*, which is consistent with a previous study in *D. officinale* [37], and the average content was approximately 0.029 mg·g^− 1^ DW. No naringenin was detected in the PLBs of *D. officinale*, indicating that PLBs cannot be used as an alternative material for naringenin extraction (Fig. 3 c). Based on similarity in naringenin content between the whole plant (0.021 mg·g^− 1^ DW) and the stem (approx 0.029 mg·g^− 1^ DW), we concluded that naringenin derives mainly from the stem. We also established that the biomass of *D. officinale* was mainly from stems (Additional file 4).

### Sequencing, assembly and Unigenes functional annotation

A total of 39.11 Gb transcriptomic data were generated using the BGISEQ-500 platform. Clean reads were assembled using Trinity software and then clustered to remove redundancy and obtain unigenes using Tgicl. We obtained 157,901 unigenes (Additional file 5). The total length, average length, N50, and GC content were 202,415, 413 bp, 1281 bp, 2194 bp, and 40.39%, respectively.

We mapped all unigenes to the major functional databases for annotation. There were 99,474 (NR: 63.00%), 96,634 (NT: 61.20%), 62,695 (SwissProt: 39.71%), 68,459 (KOG: 43.36%), 71,648 (KEGG: 45.38%), 68,281 (GO: 43.24%) and 65,117 (Pfam: 41.24%) unigenes with functional annotations (Additional file 6). Moreover, 73,300 coding sequences were detected using Transdecoder software, 36,670 simple sequence repeats (SSRs) were found distributed among 28,910 unigenes, and 2893 unigenes encoding transcription factors were predicted.

### Identification and KEGG pathway enrichment of differentially expressed genes (DEGs)

**Fig. 4:**
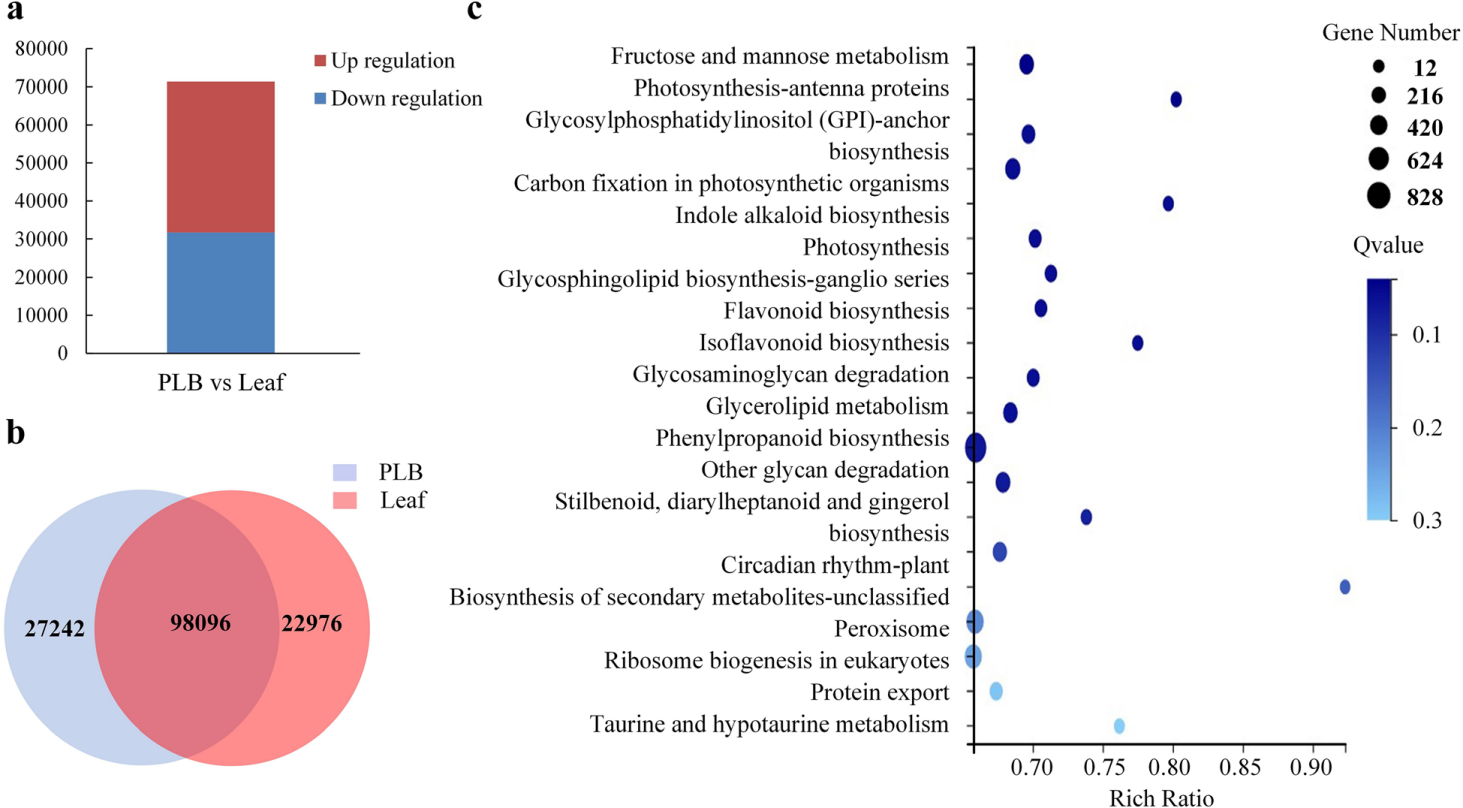
Identification of differentially expressed genes and KEGG pathway analysis. **a** Number of up-regulated and down-regulated genes in PLB compared with leaf. **b** Venn diagram showing genes expressed specifically in leaves and PLBs. **c** Bubble diagram showing enrichments of DEGs between leaves and PLBs in KEGG pathways

We quantified expression of 148,314 unigenes from the 157,901 unigenes assembled based on FPKM ≥1.0. Poisson distribution method with log_2_FC (fold change) values ≥1.0 or ≤ − 1.0 and false discovery rate (FDR) ≤ 0.001 identified 71,307 DEGs between leaf and PLB. Among these DEGs, 39,597 unigenes were up-regulated and 31,710 unigenes were down-regulated in PLBs compared with leaf (Fig. 4 a). A Poisson distribution revealed that 27,242 unigenes were specifically expressed in leaves, 11,976 unigenes were expressed only in PLBs, and 98,096 unigenes were expressed in both of these samples (Fig. 4 b, Additional file 7). The large number of DEGs identified demonstrated significant differences between leaves and PLBs, which directly reflected the critical difference in the appearance of the different tissue types.

KEGG enrichment analysis of 71,307 DEGs identified in PLBs versus leaves revealed that these genes were associated with 135 mapped pathways (Additional file 8). The genes were mainly enriched in “phenylpropanoid biosynthesis”, “flavonoid and isoflavonoid”, “carbon fixation and photosynthesis”, “fructose and mannose metabolism”, and “indole alkaloid biosynthesis”. (Fig. 4 c).

### Differential expression of genes involved in polysaccharide biosynthetic pathways

**Fig. 5:**
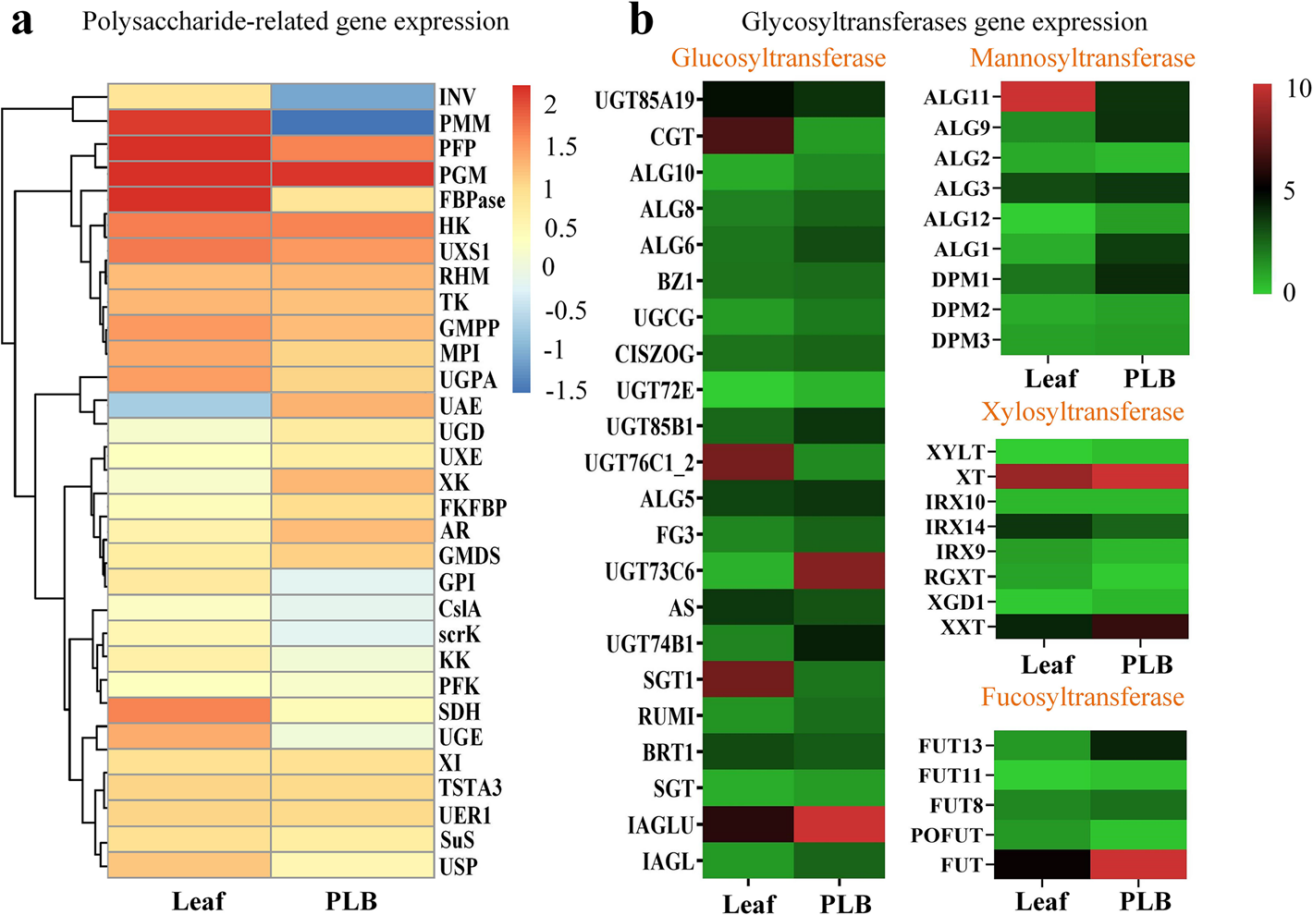
Average expression level of putative genes encoding key enzymes involved in polysaccharide biosynthesis of *D. officinale* between leaves and PLBs. **a** Heatmap showing known putative genes in polysaccharides biosynthesis. **b** Heatmap showing uncertain glycosyltransferases in DOP biosynthesis

Polysaccharides were much more abundant in leaves than in PLBs. We therefore studied the differences in expression of genes encoding enzymes involved in polysaccharide biosynthesis between PLBs and leaves (Fig. 5). Expression levels of some genes were much higher in leaves than in PLBs, such as genes encoding INV, CslA, FBPase, PFP, MPI, scrK, KK, USP, UGPA, PMM, GMPP and UXS1. A higher level of gene expression was positively correlated with a higher content of polysaccharide in leaves. Compared with PLBs, the *INV* gene was highly expressed while *SuS* gene expression was increased mildly in leaves, indicating that more sucrose was required to biosynthesize more downstream polysaccharides. Lower expression of a gene encoding CslA was positively correlated with the lower polysaccharides (mainly mannose monosaccharides) content in PLBs (Fig. 5 a).

Meanwhile, there was no significant difference in the expression of genes encoding enzymes such as PGM, HK, TK, UXE, PFK, TSTA3 and UER1 between leaf and PLB, suggesting that the catalysis reactions performed by these enzymes are not restrictive steps for DOP biosynthesis. Genes encoding enzymes such as UAE, UGD, XK, AR and GMDS were negatively correlated with DOP content, these enzymes are all bifunctional and in closed-loop pathways (Fig. 5 a). This might indicate that these bifunctional enzymes have greater ability for degradation than polysaccharide biosynthesis.

Glycosyltransferases are a very widespread group of carbohydrate-active enzymes participating in glycan and glycoside biosynthesis in higher plants [38]. We identified 553 glycosyltransferase genes, including 305 glucosyltransferase genes, 29 fucosyltransferase genes, 110 mannosyltransferase genes and 106 xylosyltransferase genes, using BLASTX in the transcriptomes. From these, 22 full-length glucosyltransferases genes, 5 fucosyltransferase genes, 9 mannosyltransferase genes and 8 xylosyltransferase genes were identified. The expression levels in leaf and PLBs of genes encoding enzymes such as CGT, UGT76C1_2, SGT1 and ALG11 were in line with the patterns of DOP content, suggesting these could be candidate genes encoding enzymes involved in DOP biosynthesis in *D. officinale* (Fig. 5 b, Additional file 9).

### Analysis of differential gene expression in alkaloids biosynthesis

**Fig. 6:**
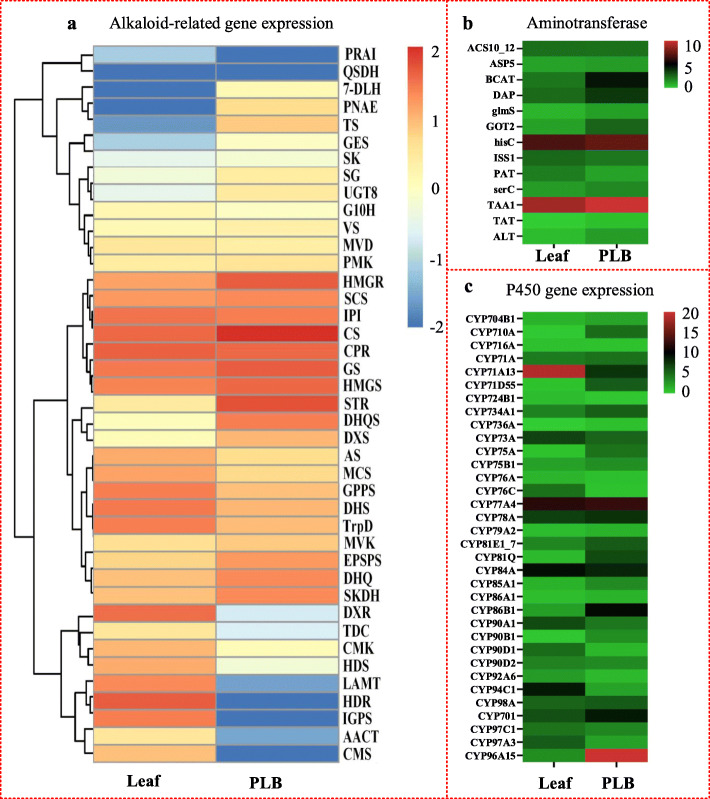
Average expression level of putative genes encoding enzymes involved in alkaloid biosynthesis in *D. officinale* between PLB and leaf. **a** Expression level variation of putative upstream and downstream genes in strictosidine biosynthesis in *D. officinale*. **b** Expression level variation among putative genes encoding aminotransferases involved in alkaloid biosynthesis in *D. officinale*. **c** Differences in expression level among putative p450 genes in alkaloids biosynthesis in *D. officinale*

A previous study showed that the alkaloids in *D. officinale* are mainly terpenoid indole alkaloids (TIAs). We analyzed differences in expression of genes encoding enzymes upstream of MVA, MEP and Shikimate pathway between leaves and PLBs. There were significant differences between leaves and PLBs, but regulatory patterns among these genes were not consistent (Fig. 6 a). Disorder of gene expression indicated that the intermediates upstream of TIA biosynthesis are not restrictive products, which was further verified by the expression of two genes encoding LAMT and TDC involved in biosynthesizing loganin and tryptamine, respectively. The content of alkaloids was higher in PLBs than in leaves, but the expression levels of genes encoding LAMT and TDC enzymes were lower in PLBs than in leaves. There was no significant difference in expression of the gene encoding SCS between PLBs and leaves; SCS catalyzes the conversion of loganin into secologanin. In conclusion, most intermediates, especially loganin and secologanin, as direct substrates for strictosidine, were sufficient for biosynthesis of specific downstream alkaloids. The expression level of the gene encoding enzyme STR was much higher in PLBs than in leaves, meaning a higher content of precursor strictosidine is necessary for producing additional alkaloids in PLBs.

In the downstream biosynthesis pathway of specific alkaloids in *D. officinale*, expression level of putative genes encoding enzymes SG, GS and PNAE were much higher in PLBs than in leaves, and the expression level of the gene encoding VS was slightly higher in PLBs (Fig. 6 a). Genes encoding SG, GS and VS were therefore predicted to be involved in *D. officinale* alkaloids biosythesis. Moreover, these predicted genes involved in TIAs biosynthesis in *D. officinale* indicate that alkaloids of *D. officinale* might include strictosidine aglycone, geissoschizine and vinorine.

Some aminotransferases and P450 superfamily enzymes are necessary for alkaloid biosynthesis. By comparing expression levels between PLBs and leaves, we found that some aminotransferases and P450s were more highly expressed in PLBs than in leaves (Fig. 6 b, c, Additional file 9). We therefore speculated that some highly expressed aminotransferases and P450s might participate in alkaloid biosynthesis in *D. officinale*.

### Differential expression of genes in the flavone biosynthetic pathway

**Fig. 7:**
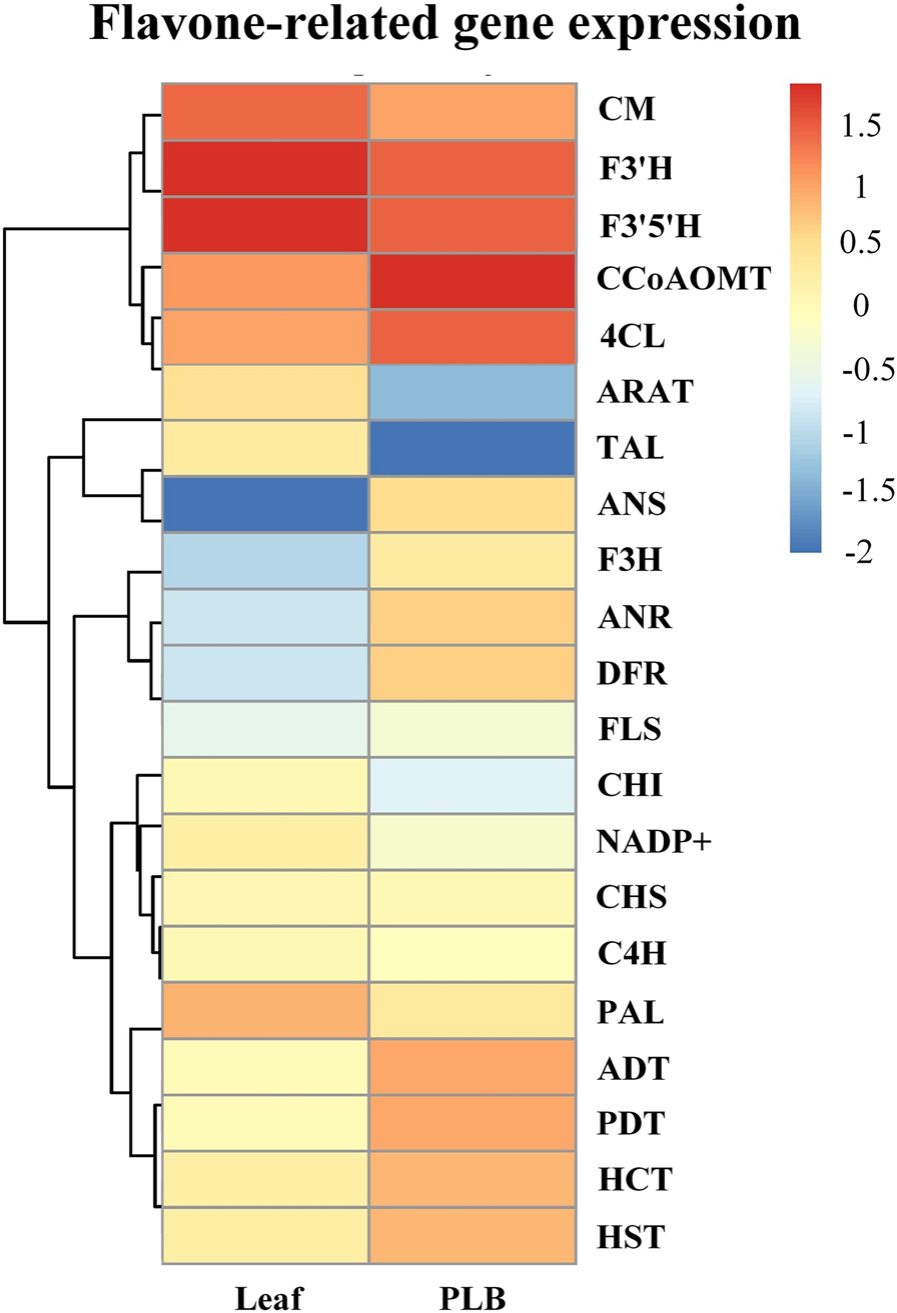
Average expression level of putative key genes encoding enzymes involved in flavonoids biosynthesis between leaves and PLBs of *D. officinale*

Expression levels of genes encoding enzymes involved in flavones biosynthesis were different between PLBs and leaves (Fig. 7). TAL and PAL were expressed more highly in leaves than in PLBs, which corresponded with the higher content of flavonoids in leaves. ADT and PDT were expressed much more highly than PAL or TAL in PLBs, suggesting that PLBs need to produce more L-phenylalanine as a substrate for downstream flavonoid biosynthesis. The lower expression levels of ANS and ANR in leaves compared with PLBs suggested low contents of cyanidin and epicatechin in leaves of *D. officinale*.

Naringenin could not be quantified in PLBs. Based on low expression of CHI, high expression of DFR, and relatively high expression of F3’H and F3’5’H in PLBs, we concluded that naringenin is an intermediate product converted to downstream metabolites and not stored in PLBs. The naringenin content in leaves of *D. officinale* was too low to be detected. However, high expression of CHI, F3’H and F3’5’H, and lower expression of DFR indicated that naringenin might be transferred to stem and converted to downstream flavonoids. Naringenin could be detected only in the stem of *D. officinale*. There is a possibility that a special organelle exists in the stem, functioning to accumulate naringenin and transfer it from the leaf to the stem in *D. officinale*. HCT and HST were more highly expressed in PLBs than in leaves, suggesting that most of the eriodictyol in PLBs comes from caffeoyl-CoA, which is consistent with relatively lower expression of F3’H and F3’5’H, and low naringenin content in PLBs.

### Validation of genes expression levels by real-time fluorescent quantitative PCR (RT-qPCR)

**Fig. 8:**
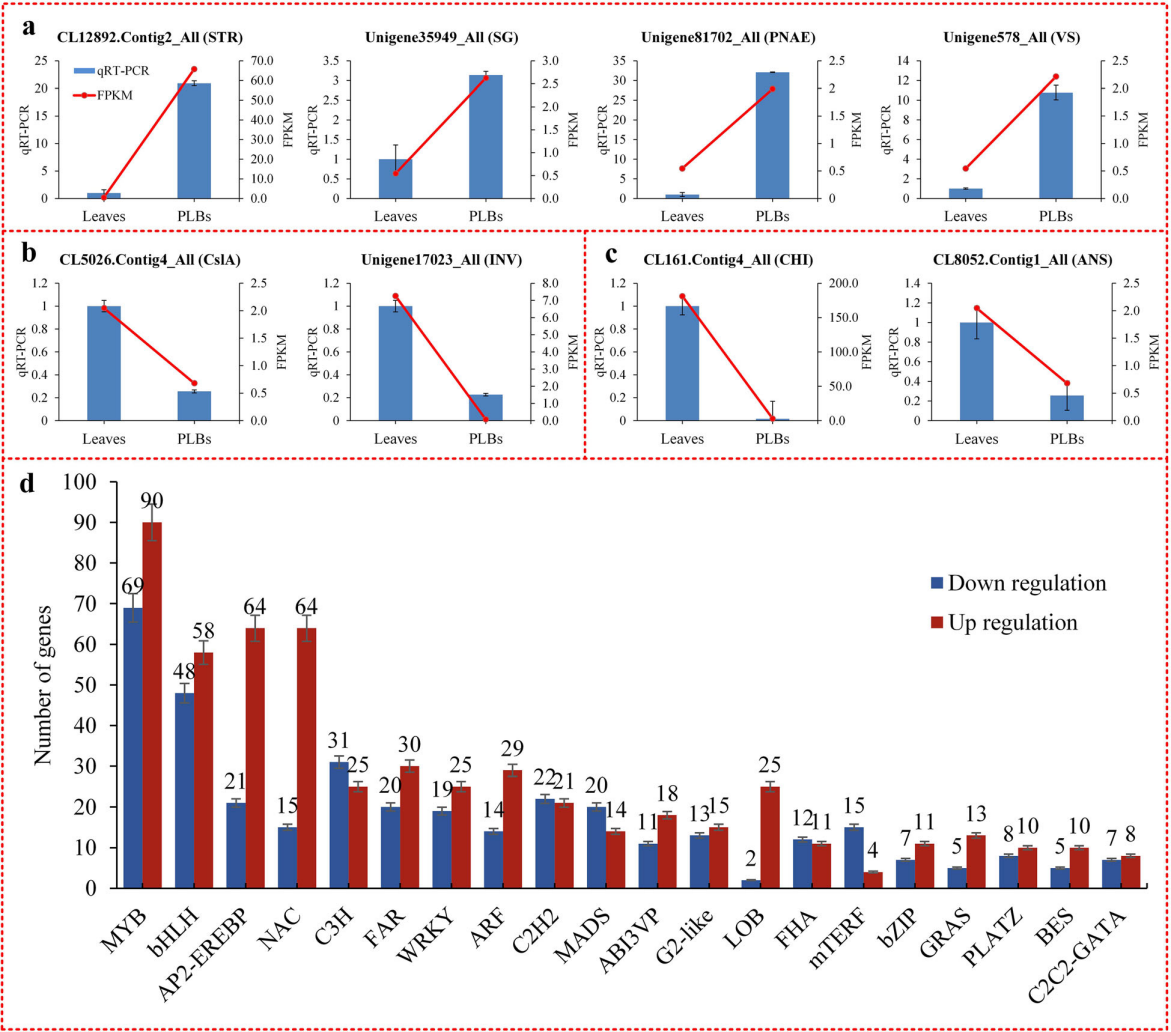
RT-qPCR analysis and number of differentially expressed transcription factors. RT-qPCR analysis of genes encoding enzymes involved in alkaloid biosynthesis (**a**), polysaccharide biosynthesis (**b**), and flavonoid biosynthesis (**c**). The number of major transcription factor genes up-regulation and down-regulation of in PLBs comparing to leaves of *D. officinale* (**d**)*.* Subunit 5.8S of rRNA was used as an internal gene for normalization. PLB and leaf samples were used as normalizers in each experiment. Blue bars represent RT-qPCR data, and red lines indicate FPKM values from RNA-seq. Data represent mean ± standard error of three replicates. The left Y-axis denotes relative expression levels of genes determined by RT-qPCR, and the right Y-axis denotes the FPKM values of RNA-Seq data

We verified the expression levels of genes encoding enzymes such as STR, SG, PNAE, VS, CslA, CHI, INV and ANS using RT-qPCR analysis (Fig. 8 a, b, c). Expression levels of genes encoding enzymes involved in alkaloids biosynthesis were higher in PLBs than in leaves (Fig. 8 a). Meanwhile, expression levels of those genes encoding enzymes in polysaccharide and flavonoid biosynthesis were higher in leaves than in PLBs (Fig. 8 b, c). These results from RT-qPCR analysis were consistent with the data from RNA-seq analysis.

### Detection of transcription factor families

Transcriptional factors (TFs) play an important role in the regulation of secondary metabolites by coordinating the expression of biosynthetic genes and controlling the production of secondary metabolites in plants [39]. We identified 1113 unique putative transcripts encoding TFs differentially expressed between PLBs and leaves, which belonged to 51 known TF families (Additional file 10). Most belonged to the MYB, AP2/EREBP, bHLH and NAC families. The number of up-regulated and down-regulated TFs of each type is shown in Fig. 8 d, and a striking number of differentially expressed TFs were identified between PLBs and leaves.

## Discussion

Extraction method is reported to have little effect on the types of monosaccharide, but to influence the monosaccharide composition of heterogeneous polysaccharides [40]. The diffusion rate of polysaccharides in the HWE method night increase dramatically owing to the raised temperature of the water, improving the extraction efficiency [41]. Our results indicates that the HWE method is more suitable than the UAE method for DOPs extraction, consistent with this conclusion.

Among organs of *D. officinale,* the stem had the highest content of polysaccharides—up to 402.93 mg·g^− 1^ DW. This is consistent with the findings of previous researchers in *D. officinale* [22]. By contrast, PLBs had a lower content of polysaccharides, indicating that the PLBs cannot be used as a substitute for plant organs in producing DOPs.

In spite of the relatively low content of total alkaloids in *D. officinale*, more and more attention has been paid to their broad pharmacological activities, such as antipyretic, analgesic and anti-tumor effects [12]. PLBs were much more suitable for producing alkaloids in our study, providing a new strategy for future large-scale production of alkaloids in *D. officinale.*

KEGG enrichment analysis indicated that comparative RNA-Seq between leaves and PLBs was consistent with the contents of DOPs, alkaloids and flavonoids between PLBs and leaves of *D. officinale*. It also demonstrated our reasonable prediction of the putative biosynthesis pathway of DOPs, alkaloids and flavonoids in *D. officinale*.

Characterization of the correlation between the content of DOPs, alkaloids and flavonoids and comparative transcriptome sequence results allowed discovery of many putative genes encoding enzymes involved in biosynthesis of DOPs, alkaloids and flavonoids in *D. officinale*. Next, we will clone of the genes encoding enzymes such as SG, GS, PNAE and VS from *D. officinale* according to orthologous sequences from RNA-seq and construct the expression vectors to express recombinant proteins. The enzyme activities of these recombinant proteins in vitro will be assessed to validate their function in alkaloid biosynthesis. The expression patterns and substrate specificity of these putative enzymes and their subcellular localization will also need to be studied.

Previous reports indicate that TFs play important roles in regulating alkaloid biosynthesis, such as members of the apetala2/ethylene response factor (AP2/ERFs) [42, 43], C2H2 zinc fingers [44], WRKY [45] and basic helix-loop-helix (bHLH) [46] families that are involved in regulating TIA biosynthesis. ORCA3, an AP2/ERF TF, induces the expression of a number of TIA biosynthetic genes such as *G10H*, *CPR*, *STR*, *AS*, *TDC*, and *DXS* in the vindoline or tryptamine branch pathway [47, 48]. CrMYC, belonging to the bHLH family, activates ORCA3 and then induces expression of several TIA biosynthetic genes, such as *TDC* and *CPR* [49]. CrBPF1 is a MYB transcription factor that regulates the expression of *STR* in *C. roseus* [50]. In our study, we identified TFs such as MYB, AP2/EREBP, bHLH and NAC to be involved in alkaloids biosynthesis in *D. officinale*.

## Conclusions

The best protocol for PLB induction in *D. officinale* uses seed as explants and 1/2 MS + α-NAA 0.5 mg·L^− 1^ + 6-BA 1.0 mg·L^− 1^ + 2, 4-D 1.5–2.0 mg·L^− 1^ + potato juice 100 g·L^− 1^ as the full medium.

The distribution of polysaccharides, alkaloids and flavonoids among the organs of *D. officinale* plant was clarified in this study. Contents of polysaccharides, alkaloids and flavonoids are highest in stems, PLBs and leaves of *D. officinale*, respectively. Hot water extraction (HWE) is a better method than UAE for polysaccharides extraction from *D. officinale*. PLBs can be used as an alternative for producing alkaloids in *D. officinale*. However, naringenin is produced exclusively in stems, with no detectable amount in PLBs.

Comparative RNA-Seq analysis of *D. officinale* PLBs and leaves enabled identification of candidate genes encoding enzymes involved in polysaccharide, alkaloid and flavonoid biosynthesis. We report on genes encoding enzymes such as SG, GS and VS in alkaloids biosynthesis of *D. officinale* for the first time to our knowledge. Moreover, some TFs involved in regulating alkaloid biosynthesis are differentially expressed in leaves and PLBs of *D. officinale*.

## Methods

### Plant materials and reagents

Plants were from Huoshan County, Anhui Province, China, identified as *Dendrobium officinale* Kimura et Migo. They were grown at 25 ± 2 °C during the day and 23 ± 2 °C at night with 60–70% relative humidity and a light/dark cycle of 14/10 h in the greenhouse of Anhui University of Chinese Medicine, Hefei, China. A specimen of *D. officinale* used in this study has been deposited in the Herbarium of Anhui University of Chinese Medicine with the depository no. is ACM025738.

Standards of (+)-glucose, dendrobine, rutin and naringenin (> 98% purity) were purchased from Chengdu Push Bio-technology CO., Ltd. Methanol (HPLC grade) was purchased from Oceanpak, and CTAB-pBIOZOL reagent used for total RNA extraction was purchased from Bioflux (Beijing, China). HPLC-grade water was prepared using a water purification system from Pall Filter Co., Ltd. (Beijing, China). Plant growth regulators (PGRs) including 6-BA, α-NAA and 2, 4-D were purchased from Solarbio. Agents for induction and propagation of PLBs were purchased from Sinopharm group, and all of the other agents were analytical grade.

### Induction and propagation of *D. officinale* Protocorms and PLBs

*D. officinale* capsules approaching maturity, leaves, stem tips, stem fragments, and stems with nodes were collected and sterilized to induce protocorms and PLBs (capsules were punctured to release seeds as explants on medium under sterile condition); each explant was prepared with more than 30 replicates. Explants were placed on 1/2 MS medium + 2, 4-D 0.5 mg·L^− 1^ + 30 g·L^− 1^ sucrose + 7 g·L^− 1^ agar at pH 5.6 to 5.8, and placed in a tissue culture room at 70% relative humidity, 1600 lx illumination and 16/8 h light/dark for 60 days to identify the optimal explants for protocorms and PLBs induction.

Protocorms and PLBs induced as above were collected as explants to be cut into pieces of approximately 0.5 cm × 0.5 cm to proliferate more PBLs, and the optimum culture protocol for proliferation of *D. officinale* PLBs was screened. An L_16_(4^5^) orthogonal experiment without regarding interactions among factors was performed; the factors and their levels were selected on the basis of previous experiments and reports [51]. Four different concentrations of MS medium were used as test media (Factor A): 1/4MS, 1/2MS, MS and 2MS. Different concentrations of plant growth regulators were arranged in different combinations to screen the optimal combination: α-NAA (Factor B) (0.1, 0.2, 0.5 and 1.0 mg·L^− 1^), 6-BA (Factor C) (0.5, 1.0, 1.5 and 2.0 mg·L^− 1^) and 2,4-D (Factor D) (1.5, 2.0, 2.5 and 3.0 mg·L^− 1^). Explants in tissue culture with natural additives and fruit juice grew much better than those without natural additives and fruit juice [52, 53]. Therefore, for the PLBs proliferation process, potato juice was selected as an additive to accelerate PLB growth and propagation. To prepare this additive, 200 g fresh potato was chopped into small pieces, mixed with purified water to 1 L and boiled to a mush. The mushy potato was passed through gauze to collect the juice as additive. Concentrations of potato juice (Factor E) used were 50, 100, 150 and 200 g·L^− 1^. All test media for PLB proliferation included basic medium, plant growth regulators, potato juice, sucrose (30 g·L^− 1^) and agar (7 g·L^− 1^) at pH 5.6 to 5.8; the design of the orthogonal experiment table head is shown in Additional file 11. Each group comprised 10 bottles with 4 explants, and culture conditions were light/dark for 16/8 h per day, at 1600 lx and 25 (± 1) °C. Observations and statistical analysis were performed to analyze the propagation coefficient 2 months after inoculation. The propagation coefficient was calculated as follows: Propagation coefficient (%) = (FW_1_ – FW_0_)/ FW_0_ × 100%; FW0 and FW1 are the fresh weight before inoculation and after culture, respectively.

### Ultrasound-assisted extraction (UAE) and hot water extraction (HWE) of Total polysaccharides

Stems, leaves, roots and whole plants from approximately 3-years-old *D. officinale* and PLBs were harvested, cleaned and dried in an oven at 50 °C to a constant weight. Samples were ground to a fine powder using a pulverizer and sieved through an aperture of 40 mesh sieve. The water-soluble polysaccharides in *D. officinale* were extracted using the UAE method [41] and HWE method [8] with a few modifications. This allowed selection of the better technique for extraction of polysaccharides from *D officinale*.

The modified UAE method was as follows: 10 mL of double-distilled water was added to each 0.2 g pulverized sample, and the samples were homogenized in an ultrasonic cleaner at 40 °C, 40 Hz for 0.5 h and filtered through filter-paper to obtain the filtrate. The above steps were repeated once, and all filtrate was collected. The filtrate was concentrated at 55 °C until its volume was reduced to 10 mL. Next, 40 mL of ethanol was added into the concentrated filtrate, which was centrifuged at 4000 g for 5 min, and the supernatant was discarded. The resulting samples were dissolved in 25 mL of pure water and 25 mL of Savage reagent was added to remove impurities such as protein and nucleic acid. Sample were centrifuged at 1000 g for 5 min, and 20 mL of the supernatant was transferred into a 50 mL volumetric flask and mixed with pure water to a constant volume of 50 mL for polysaccharide extraction.

The HWE method with a few modifications was as follows: 0.3 g sample was placed in a round-bottomed flask, and 200 mL water was added. The sample solution was then heated and refluxed for 3 h. The reflux extract was cooled to room temperature before filtering. The filtrate was transferred into a 250 mL volumetric flask and mixed with double-distilled water to a constant volume of 250 mL. Then, 2 mL of sample solution was taken into a 15 mL centrifuge tube and 10 mL of solute ethanol was added, shaken and refrigerated for 1 h followed by centrifugation at 1000×g for 20 min. The supernatant was discarded, and the precipitate was washed twice with 8 mL of 80% ethanol. The final precipitate was dissolved in heated water and transferred to a 25 mL volumetric flask. The solution was cooled before mixing it with pure water to a constant volume of 25 mL for polysaccharide extraction.

### Determination of Total polysaccharides

A sample (1.0 mL) of each polysaccharide extract was transferred into a 10.0 mL test tube, then 1.0 mL of 5% phenol was added and vortexed quickly. The solution was mixed thoroughly before adding 5.0 mL of concentrated sulfuric acid, shaking and placing in a water bath at 100 °C for 20 min. The solution was then placed in an ice bath to cool for 5 min. The absorbance of the sample solution was measured at a wavelength of 488 nm using an ultraviolet/visible spectrophotometer with 1 mL of water as a blank; parallel detection was conducted three times. A standard curve was prepared from the D-glucose reference. Each sample was assayed three times, and the polysaccharide content was expressed as the weight of polysaccharides to the dried weight of starting materials.

### Extraction and determination of Total alkaloids

Sample powder (0.5 g) was placed into a 125 mL ground-mouth flask and mixed with 30 mL of petroleum ether. The sample mixture was placed in a constant temperature water bath (35 °C) to degrease for 30 min, then the supernatant was removed and the petroleum ether evaporated. The pH was adjusted with ammonium hydroxide, and 10.0 mL of chloroform was added. The solution was then refluxed for 2 h in a water bath at 80 °C and allowed to cool for 20 min at room temperature before filtering. The filtrate was obtained as total alkaloids solution.

Total alkaloids solution (5.0 mL) was mixed with 5 mL of chloroform, then 5.0 mL of pH 4.5 buffer and 2.0 mL 0.04% bromocresol green solution were added sequentially. The mixture was shaken vigorously for 3 min and let stand for 30 min before filtering. The filtrate (5.0 mL) was mixed with 1.0 mL 0.01 M NaOH-ethanol solution and vortexed. The absorbance of the samples was measured at 620 nm using a UV-visible spectrophotometer with chloroform as blank. A standard curve was prepared using dendrobine as reference (> 98% purity; bought from Chengdu Push Bio-technology Co., Ltd). (0.857, 1.714, 2.571, 3.429, 4.286, and 5.143 μg·mL^− 1^). Parallel detection was carried out three times, and the alkaloid content was shown as the weight of total alkaloids to the dried weight of starting materials.

### Extraction and determination of Total flavonoids

Sample powder (1.00 g) was dissolved in 50 mL of 70% ethanol and refluxed at 60 °C for 2 h, then filtered. The filtrate was mixed with 70% ethanol again to a constant volume of 50 mL to yield the total flavonoid extracts.

Total flavonoid extract (1.0 mL) was mixed with 5.0 mL of 70% ethanol, then 1.0 mL of 5% NaNO_2_ was added and mixed well. After standing for 6 min, 1.0 mL of 10% Al (NO_3_)_3_ was added and mixed well. The solution was allowed to stand for a further 6 min before adding 10 mL of 1 M NaOH and then mixing with 70% ethanol to 25 mL and standing for 15 min. The absorbance of the sample solution was measured at 510 nm using an ultraviolet/visible spectrophotometer. A standard curve was prepared from rutin as reference (2.208, 4.416, 6.624, 8.832, and 11.040 μg·mL^− 1^). Each sample was assayed three times, and the flavonoid content was shown as the weight of total flavonoids to the dried weight of starting materials.

### Extraction and quantification of Naringenin

Naringenin was extracted using optimized methods: methanol (20 mL) mixed with 20% hydrochloric acid (5.0 mL) was used as solvent; particle size of dry powder of *D. officinale* was < 0.355 mm; temperature was 80 °C; condensation reflux extraction time was 90 min; supernatant (20 mL) was evaporated using a rotating evaporator until dry. Methanol (5.0 mL) was added to dissolve naringenin, and the solution was filtered through a 0.22 mm nylon membrane filter before HPLC analysis.

HPLC analysis was performed on an Agilent HPLC system, including quaternary solvent management, sampler manager, separation system and detection systems. An Agilent C_18_ column (4.6 mm × 250 mm, 5 μm) was used at a column temperature of 30 °C. Standards and samples were separated using a gradient mobile phase consisting of 0.2% phosphate buffer (A) and methanol (B). The gradient elution program was as follows: 0.0–5.0 min, 0–25% B; 5.0–10.0 min, 25–30% B; 10.0–25.0 min, 30–40% B; 25.0–45.0 min, 40–55% B; 45.0–50.0 min, 55–60% B. The flow rate was 1 mL·min^− 1^, and naringenin was detected at 280 nm. The injection volume was 10 μL.

Naringenin stock solution was prepared and diluted to an appropriate concentration for preparation of a calibration curve. The calibration curve was prepared according to linear plots of naringenin concentration versus the corresponding chromato-graphic peak area.

### Total RNA extraction, construction of cDNA libraries and RNA-Seq

The ethanol precipitation protocol and CTAB-pBIOZOL reagent were used to purify total RNA from PLBs and leaves according to the manufacturer’s instructions (Bioflux), and each sample was prepared with three biological replicates. Tissue samples (80 mg) were ground into powder in liquid nitrogen and the powdered samples were transferred into 1.5 mL of preheated 65 °C CTAB-pBIOZOL reagent. The sample solution was incubated in a thermostatic mixer at 65 °C for 15 min to completely dissociate the nucleoprotein complexes. The supernatant was obtained by centrifuging the solution at 12,000×g, at 4 °C for 5 min. The supernatant was mixed with 400 μL of chloroform (per 1.5 mL of added CTAB-pBIOZOL reagent), and the sample mixture was centrifuged at 12,000×g, 4 °C for 10 min. The supernatant was transferred to a new 2 mL tube, and 700 μL of acidic phenol and 200 μL of chloroform were added followed by centrifugation at 12,000×g, 4 °C for 10 min. The aqueous supernatant was collected, and an equal volume of chloroform was added into it followed by centrifugation at 12,000×g, 4 °C for 10 min. An equal volume of isopropyl alcohol was added into the supernatant, and the mixture was placed at − 20 °C for 2 h to precipitate before centrifuging at 12000×g, 4 °C for 20 min; and the supernatant was removed. The RNA pellet was washed with 1 mL of 75% ethanol before being air-dried in a biosafety cabinet and dissolved in 50 μL of DEPC-treated water. Subsequently, quality control and quantification of total RNA were performed using a Nano Drop and Agilent 2100 bioanalyzer (Thermo Fisher Scientific, MA, USA).

Oligo (dT)-attached magnetic beads were used to purify mRNA. The purified mRNA was split into small pieces using fragment buffer at an appropriate temperature. Random hexamer-primed reverse transcription was performed to synthesize first-strand cDNA, followed by second-strand cDNA synthesis. A-Tail Mix and RNA Index Adapter were then added to repair the ends. The cDNA fragments obtained were amplified by PCR. The products were purified using Ampure XP Beads and dissolved in EB solution. Products were verified for quality control using an Agilent 2100 bioanalyzer. Double-stranded PCR products were denatured and cycled using the splint oligo-nucleotide sequences to obtain the final library in single-stranded circular DNA (ssCir DNA) format. The library was amplified with phi29 to obtain DNA nanoballs (DNBs), each molecule of which had over 300 copies. The DNBs were added into the patterned nanoarray, and 100 base pairs of reads was generated on the BGIseq500 platform.

### Functional annotation of Unigenes using a reference genome

Original sequencing data (original polymerase reads) produced from raw data generated by the Pacific Biosciences Sequel system were processed using the SMRT analysis package version 2.3.0 according to the IsoSeq protocol (Pacific Biosciences, https://www.pacb.com/products-and-services/analytical-software/smrt-analysis). ROIs (reads of insert) were generated from original polymerase reads having full passes > 0 and the predicted consensus accuracy > 0.75. According to whether the primers were 5′ primers, 3′ primers or poly-A tails, ROIs with a minimum length of 300 bp were divided into non-full-length and full-length transcribed sequences. The full-length sequences were processed to de novo consensus isoforms using the ICE (Iterative Clustering for Error Correction) algorithm and then polished using the Quiver quality-aware algorithm. High quality de novo consensus isoforms (expected Quiver accuracy ≥0.95) from each library were combined, and then redundancy was removed using CD-HIT [54] founded on the sequence similarity to get final, unique, full-length isoforms.

Final, complete subtypes were mapped to SwissProt (manually annotated and reviewed protein sequence database), NR (NCBI non-redundant protein sequence), KEGG (Kyoto Encyclopedia of Genes and Genomes), NT (NCBI non-redundant nucleotide sequence) and KOG (Clusters of Eukaryotic Orthologous Groups) databases using BLAST software (version 2.2.23) [55] with default parameters (under a threshold E-value ≤10–5) to obtain isoform annotations. GO (Gene Ontology) annotations and functional classifications were acquired using the Blast2GO program (version 2.5.0, E-value ≤10–5) [56] according to NR annotations. InterProScan5 software (version 5.11–51.0) [57] was used to acquire annotations from the InterPro database.

### Identification of differentially expressed genes (DEGs)

All clean reads were mapped to the full-length reference transcriptome using BLAST software. Gene expression levels were determined by the number of full-length transcripts belonging to a cluster after ice clustering and the CD-HIT process. Total isoforms counts were used to normalize the counts in each sample. DEGs were acquired using DEseq2 with Q value (adjusted *P* value) < 0.001 and fold change (FC) ≥ 2 or ≤ − 2 [58]. These DEGs were then carried into GO and KEGG enrichment with Phyper in the R package using Q value ≤0.05 as default.

### Comparative analysis of putative gene expression in metabolite biosynthesis pathways

The amino acid sequences of putative genes involved in the polysaccharide, alkaloid and flavonoid biosynthetic pathways were identified in the National Center for Biotechnology Information (NCBI) database (Additional file 12). All amino acid sequences of putative genes were mapped to the protein information from RNA-Seq using BLAST software to obtain the average FPKM value of each gene in each sample. Heatmaps of putative genes involved in polysaccharide, alkaloid and flavonoid biosynthesis were drawn based on FPKM value. Differences in expression levels of putative genes encoding enzymes involved in alkaloid, polysaccharide and flavone biosynthesis between PLBs and leaves were measured using the value of diffexp_log2fc_Cas-vs -Ctr (Cas is PLB, Ctr is leaf) for unigenes with high amino acid sequence identity from the RNA-seq database.

### Real-time fluorescent quantitative PCR (RT-qPCR) analysis

To verify RNA-Seq data, RT-qPCR analysis was conducted using a SuperReal PreMix Plus SyBr Green PCR kit (Qiagen) on a Cobas z480 Real-Time PCR System. Randomly selected candidate primers were designed using Primer Premier (version 5.0) (Additional file 13). Reactions contained 2.0 μl of diluted cDNA, 0.6 μl of each primer, 10 μl of 2 × SuperReal PreMix Plus, and 6.8 μl of RNase-free double-distilled water (ddH_2_O). All RT-qPCRs were performed as follows: denaturation at 95 °C for 15 min, followed by 45 cycles of 95 °C for 10 s, 58 °C for 20 s and 72 °C for 30 s. Successive RT-qPCR assays were performed using three biological replicates; to verify product specificity, melting curve analysis was performed after each amplification. A housekeeping gene (5.8S) was used as a reference, and the relative expression level of each gene was calculated using the 2^−ΔΔCt^ approach.

#### Statistical analysis

Experimental data are expressed as the mean ± standard deviation of three independent biological replicates. Statistical differences between samples were analyzed using two-way analysis of variance by SPSS (version 17.0). Values at *P* < 0.01 were considered statistically significant.

## Supplementary Information

**Additional file 1 **Putative biosynthetic pathway of polysaccharides in *D. officinale*.

**Additional file 2 **Putative biosynthetic pathway of the alkaloids in *D. officinale*.

**Additional file 3 **Putative biosynthetic pathway of the flavonoids in *D. officinale*.

**Additional file 4 **Contents of polysaccharides, alkaloids, flavonoids and naringenin among organs and PLBs of *D. officinale*.

**Additional file 5.** Core table of transcriptome sequencing.

**Additional file 6.** Genes annotation by mapping to databases.

**Additional file 7.** Information for differentially expressed genes.

**Additional file 8.** Pathway enrichment analysis of differential expressed genes.

**Additional file 9.** Putative genes involved in downstream pathways of polysaccharides and alkaloids biosynthesis.

**Additional file 10.** Classification of differentially expressed transcription factors.

**Additional file 11.** Table head design of L_16_(4^5^) orthogonal experiment for PLB proliferation.

**Additional file 12.** Enzymes involved in polysaccharide, alkaloid and flavonoid biosynthesis encoded by putative genes in corresponding pathways.

**Additional file 13.** Gene descriptions and primers used for RT-qPCR.

## Data Availability

The datasets supporting the conclusions of this article are available in the Sequence Read Archive (SRA) database of NCBI with the number GSE155403 (https://www.ncbi.nlm.nih.gov/geo/query/acc.cgi?acc=GSE155403). All of the data have been released.

## Abbreviations

TCM: Traditional Chinese Medicine
DOPs: *Dendrobium officinale* polysaccharides
Glc-1P: Glucose 1-phosphate
NDP-sugars: Nucleotide-diphospho-sugars
GTs: Glycosyltransferases
TIAs: Terpenoid indole alkaloids
PLBs: Protocorm-like bodies
DEGs: Differentially expressed genes
PGRs: Plant growth regulators
UAE: Ultrasound-assisted extraction
HWE: Hot water extraction
NCBI: National Center for Biotechnology Information
RT: Retention time
INV: β-fructofuranosidase
HK: Hexokinase
PGM: Phosphoglucomutase
XI: Xylose isomerase
AR: Aldose reductase
SDH: Sorbitol dehydrogenase
KK: Ketohexokinase
TK: Triokinase
PFP: Diphosphate-dependent phosphofructokinase
PFK: 6-phosphofructokinase
FBPase: Fructose-1,6-bisphosphatase
XK: Xylulokinase
UGE: UDP-glucuronate 4-epimerase
UXS1: UDP-glucuronate decarboxylase
UGPA: UTP-glucose-1-phosphate uridylyltransferase
UXE: UDP-arabinose 4-epimerase
UER1: 3,5-Epimerase/4-reductase
GGT: UDP-galacturonate decarboxylase
RHM: UDP-glucose 4, 6-dehydratase
scrK: Fructokinase
MPI: Mannose-6-phosphate isomerase
PMM: Phosphomannomutase
GMPP: Mannose-1-phosphate Guanylyl transferase
GMDS: GDP-mannose 4, 6-dehydratase
TSTA3: GDP-l-fucose synthase
UAE: UDP-glucuronate 4-epimerase
UGD: UDP-glucose 6-dehydrogenase
GGP: Glucose-1-phosphate guanylyltransferase
FKFBP: Fructose-2,6-bisphosphatase
SuS: Sucrose synthase
RMD: GDP-4-dehydro-D-rhamnose reductase
PGI: Glucose-6-phosphate isomerase
CslA: Cellulose synthase-like A
USP: UDP-sugar pyrophosphorylase
AACT: Acetyl-CoA acetyltransferase
HMGS: HMG-CoA synthase
HMGR: HMG-CoA reductase
MVK: Mevalonate kinase
PMK: Phosphomevelonate kinase
MVD: Mevalonate diphosphase decarboxylase
IPI: IPP isomerase
DXS: 1-deoxyxylulose-5-phosphate synthetase
DXR: 1-deoxy-D-xylulose-5-phosphate reductoisomerase
CMS: 4-diphosphocytidyl-2C-methyl- Derythritol synthase
CMK: 4-diphosphocytidyl-2C-methyl-D-erythritol kinase
HDR: 4-hydroxy-3- methylbut-2-en-1-yl diphosphate reductase
MCS: 2-C-methyl-D-erythritol 2,4- cyclodiphosphate synthase
HDS: 1-hydroxy-2-methyl- 2-(E)-butenyl-4- diphosphate syntfiase
GPPS: Geranyl diphosphate/farnesyl diphosphate synthase
GES: geranyl diphosphate diphosphatase
CPR: NADPH-hemoprotein reductase
G10H: Geraniol 8-hydroxylase
UGT8: 7-deoxyloganetic acid glucosyltransferase
LAMT: Loganate O-methyltransferase
7-DLH: 7-deoxyloganate 7-hydroxylase
SCS: Secologanin synthase
DHS: 3-deoxy-7-phosphoheptulonate synthase
DHQS: 3-dehydroquinate synthase
DHQ: 3-dehydroquinate dehydratase
SKDH: Shikimate dehydrogenase
QSDH: Quinate/shikimate dehydrogenase
SK: Shikimate kinase
EPSPS: 3-phosphoshikimate 1-carboxyvinyltransferase
CS: Chorismate synthase
AS: Anthranilate synthase
PRAI: Phosphoribosylanthranilate isomerase
TrpD: Anthranilate phosphoribosyltransferase
IGPS: Indole-3-glycerol- phosphate synthase
TS: Tryptophan synthase
TDC: Tryptophan decarboxylase
STR: Strictosidine synthase
SG: Strictosidine β-D-Glucosidase
GS: Geissoschizine synthase
PNAE: Polyneuridine-aldehyde esterase
VS: Vinorine synthase
CM: chorismate mutase
ARAT: Aromatic-amino-acid transaminase
ADT: Arogenate dehydratase
PDT: Prephenate dehydratase
NADP^+^: Arogenate dehydrogenase
ADH: Cyclohexadienyl dehydrogenase
PAL: Phenylalanine ammonia lyase
4CL: 4-coumarate CoA ligase
TAL: Tyrosine ammonia lyase
C4H: Cinnamate 4-hydroxylase
HCT: Quinate O-hydroxycinnamoyltransferase
HST: Shikimate O-hydroxy cinnamoyltransferase
CHS: Chalcone synthase
CHI: Chalcone isomerase
DFR: Dihydroflavonol-4-reductase
F3H: Flavonoid 3-hydroxylase
F3’H: Flavonoid 3′-monooxygenase
F3’5’H: Flavanoid 3′,5′-hydroxylase
DFR: Dihydroflavonol 4-reductase
CCoAOMT: Caffeoyl-CoA O-methyltransferase
ANS: Anthocyanidin synthase
ANR: Anthocyanidin reductase
FLS: Flavonol synthase
C3’H: 5-O-(4-coumaroyl)-D-quinate 3′-monooxygenase
