# Supplementary figures and images for: Putative genes in alkaloid biosynthesis identified in Dendrobium officinale by correlating the contents of major bioactive metabolites with genes expression between Protocorm-like bodies and leaves
Source: BMC Genomics. 2021 Jul 29;22:579. doi: 10.1186/s12864-021-07887-6 (PMC8323239; doi:10.1186/s12864-021-07887-6)

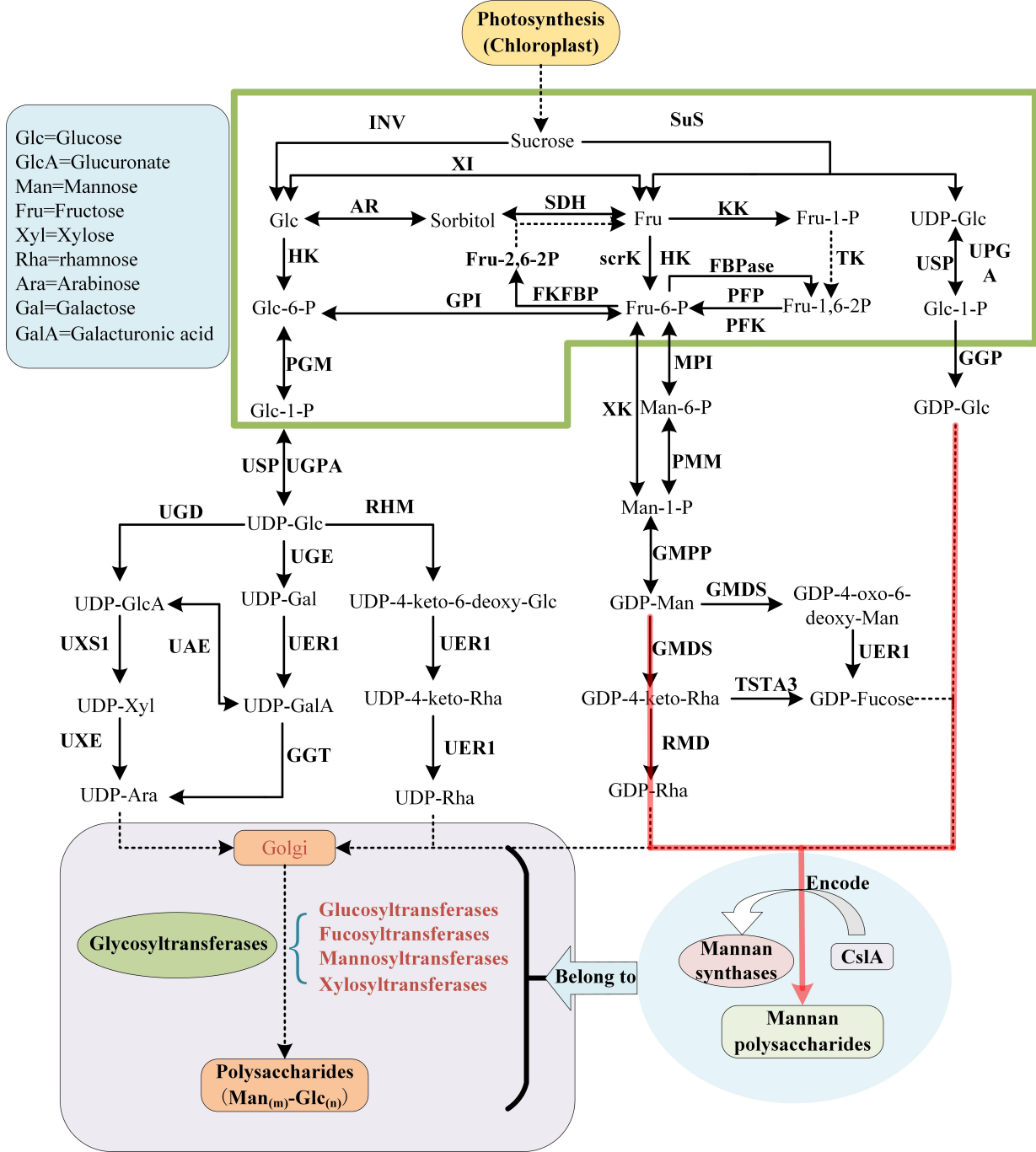

Supplement: Supplementary file 1 — Additional file 1 Putative biosynthetic pathway of polysaccharides in D. officinale. [file 12864_2021_7887_MOESM1_ESM.pdf]

## MVA Pathway

## MEP Pathway

## Shikimate Pathway

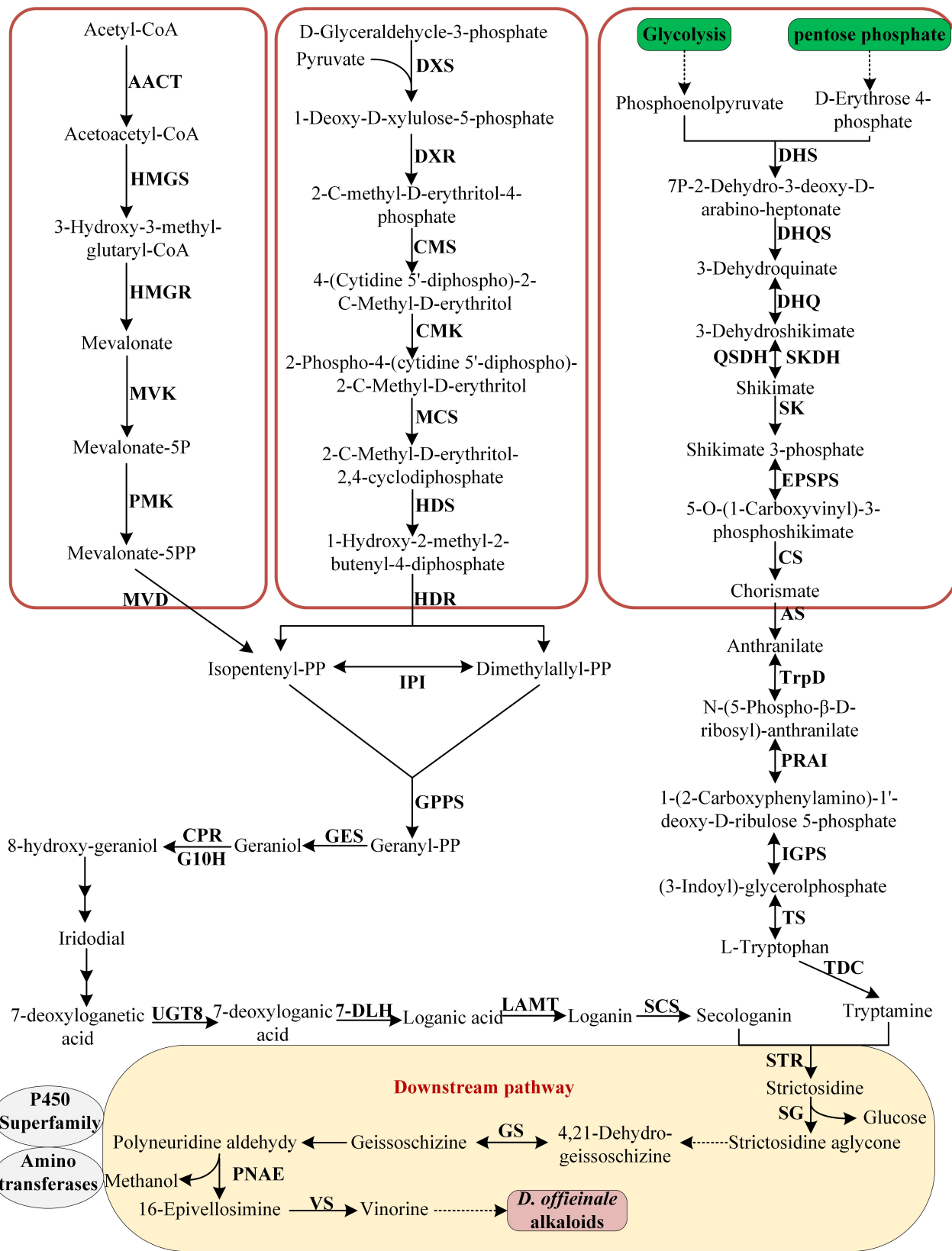

Supplement: Supplementary file 2 — Additional file 2 Putative biosynthetic pathway of the alkaloids in D. officinale. [file 12864_2021_7887_MOESM2_ESM.pdf]

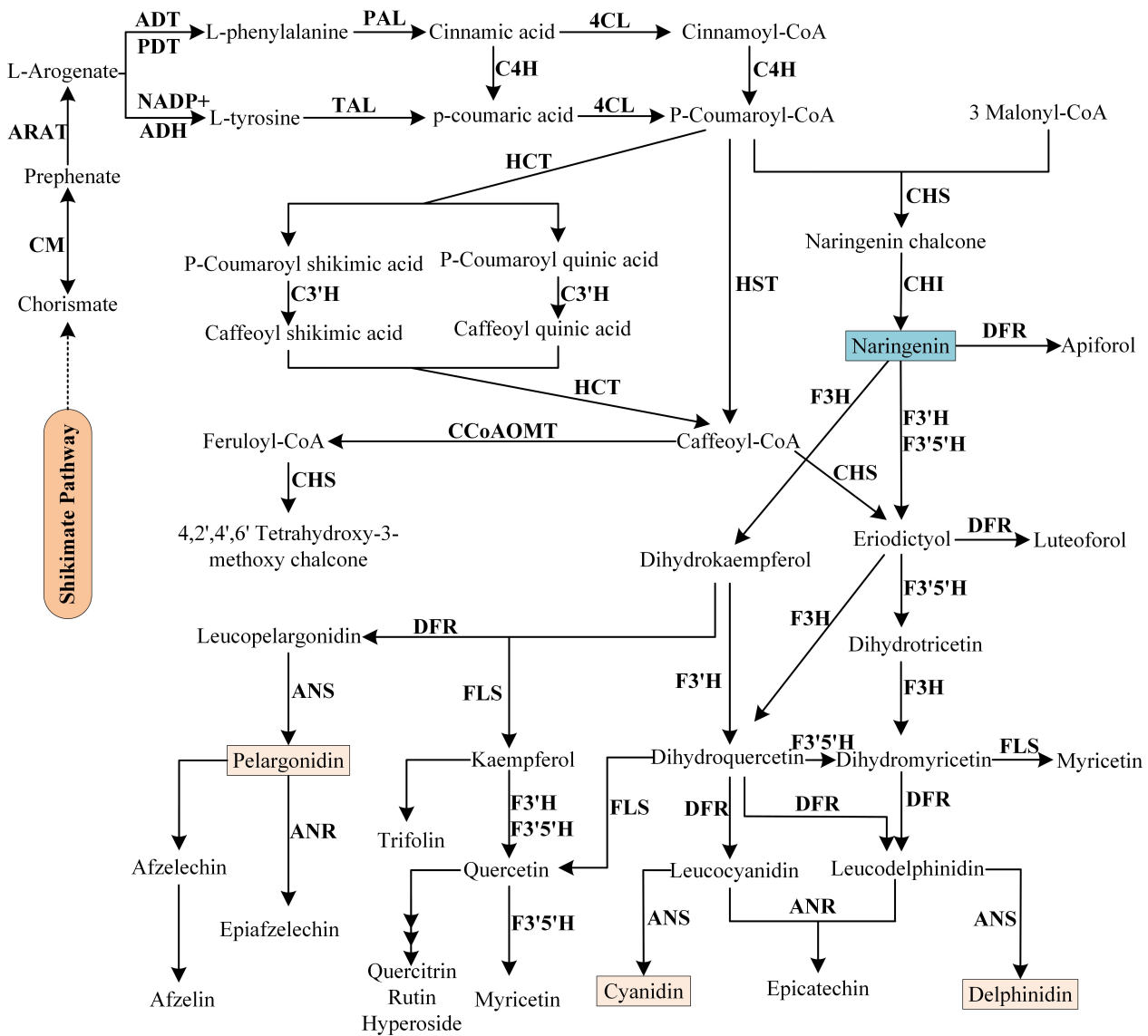

Supplement: Supplementary file 3 — Additional file 3 Putative biosynthetic pathway of the flavonoids in D. officinale. [file 12864_2021_7887_MOESM3_ESM.pdf]

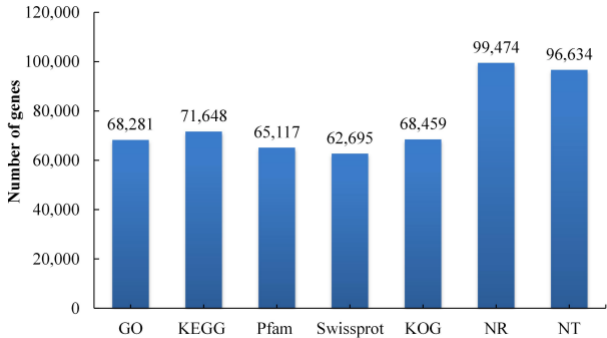

Supplement: Supplementary file 6 — Additional file 6. Genes annotation by mapping to databases. [file 12864_2021_7887_MOESM6_ESM.pdf]
